# Aberrantly DNA Methylated-Differentially Expressed Genes in Pancreatic Cancer Through an Integrated Bioinformatics Approach

**DOI:** 10.3389/fgene.2021.583568

**Published:** 2021-03-23

**Authors:** Haifeng Sun, Rui Xin, Changjun Zheng, Ge Huang

**Affiliations:** ^1^Department of Radiology, The Second Hospital of Jilin University, Changchun, China; ^2^Department of Orthopedics, The Second Hospital of Jilin University, Changchun, China

**Keywords:** pancreatic cancer, bioinformatics analysis, methylated-differentially expressed genes, prognosis, MeDEGs

## Abstract

Pancreatic cancer remains one of the chief contributors to cancer related deaths on a global scale, with its diagnosis often associated with poor prognosis and high mortality. Accumulating literature continues to highlight the role of aberrant DNA methylation in relation to pancreatic cancer progression. Integrated bioinformatics approaches in the characterization of methylated-differentially expressed genes (MeDEGs) in pancreatic cancer were employed to enhance our understanding of the potential underlying molecular mechanisms of this cancer. We initially identified differentially expressed genes (DEGs) between 178 pancreatic cancer samples and 4 normal samples and differentially methylated genes (DMGs) based on 185 pancreatic cancer samples as well as 10 normal samples by analyzing RNA sequencing data in the TCGA database. Eventually, 31 MeDEGs including 5 hypomethylated/upregulated genes and 26 hypermethylated/downregulated genes were identified. Univariate Cox model and Kaplan–Meier method revealed that, among 31 MeDEGs, 5 hypermethylated/downregulated genes (ZNF804A, ZFP82, TRIM58, SOX17, and C12orf42) were correlated with poor survival of patients with pancreatic cancer. KEGG pathway enrichment analysis by GSEA 3.0 and the protein–protein interaction (PPI) network revealed that these 5 MeDEGs were enriched in numerous cancer-related pathways in addition to interacting with each other, highlighting a significant role in the development of pancreatic cancer. Taken together, the key findings of the current study demonstrate that ZNF804A, ZFP82, TRIM58, SOX17, and C12orf42 are hypermethylated/downregulated genes in pancreatic cancer and may be associated, through their modulation of specific pathways, with unfavorable pancreatic cancer prognosis.

## Introduction

Pancreatic cancer remains one of the deadliest solid malignancies known to man, with a mortality rate comparable to its incidence. Most patients with pancreatic cancer remain asymptomatic until their disease is at an advanced stage (Wolfgang et al., 2013; Kamisawa et al., 2016). Pancreatic cancer is a particularly aggressive cancer and its diagnosis is often accompanied by an unfavorable patient outcome, most of which are the result of sporadic causes, with many modifiable risk factors such as smoking and alcohol consumption (Carrera et al., 2017). Although surgical resection and adjuvant systemic chemotherapy can offer patients with pancreatic cancer long-term survival, approximately 10–20% of patients with pancreatic cancer are suitable candidates for surgical treatment (Strobel et al., 2019). Thus, emphasizing the urgent need for more effective therapeutic strategies capable of improving the outcomes for patients with pancreatic cancer (Garrido-Laguna and Hidalgo, 2015).

Epigenetic modifications represent heritable alternations in gene expression, capable of influencing a wide array of biological processes (BPs) that play a key role in cancer progression (Oleksiewicz and Machnik, 2020). As a crucial element in epigenetics, DNA methylation regulates gene expression at a transcriptional level. The aberrant methylation of DNA regulatory region has been shown to upregulate oncogene activity and downregulate tumor suppressor gene activity without altering the sequence itself (Huang et al., 2019; Liang et al., 2019). Alterations of DNA methylation in cancer cells have been documented to play a notable role in the changes observed in the expression of genes that regulate tumor phenotypes (Cock-Rada and Weitzman, 2013). Research into gene expression and DNA methylation has provided some valuable insight for the identification of molecular markers for pancreatic cancer (Goggins, 2005). This being said, there remains insufficient study into methylation in gene expression regulation. Identification of methylation-regulated differentially expressed genes (MeDEGs) based on high-throughput data has been speculated to be of notable significance due to its potential aid in elucidating the effect of methylation in addition to identifying future research candidates (Liang et al., 2019).

Significant research has been conducted to identify MeDEGs and their relationship with pancreatic cancer using bioinformatics analysis in the present study. The Cancer Genome Atlas (TCGA) represents one of the most successful cancer genome projects to date, providing genomic sequence, expression, methylation, and copy number variation data for more than 11,000 individuals representing no less than 30 different types of cancer (Wang et al., 2016). The TCGA database possesses the capacity to screen MeDEGs using *R/Bioconductor packages edgeR* and *limma* (Zhang et al.,2019a,b). As biological system database, the Kyoto Encyclopedia of Genes and Genomes (KEGG) can integrate genomic, chemical as well as system function information (Kanehisa et al., 2008). The gene ontology (GO) is a resource that offers information about gene function by using ontologies to represent biological knowledge. These ontologies cover three domains including cellular component (CC), molecular function (MF), and BP (Ding et al., 2018). The KEGG pathway along with GO enrichment analysis were employed to identify cancer-associated GO terms and pathways where MeDEGs are significantly enriched (Gao et al., 2019). Gene set enrichment analysis (GSEA) represents a frequently used tool capable of ascertaining potential BP using their gene expression phenotype, which determines the enrichment of annotated gene sets that represent the biological function of differentially expressed genes (DEGs) in clinical samples (Han et al., 2019).

Differentially expressed genes and differentially methylated genes (DMGs) were screened from the TCGA database. The intersection between DEGs and DMGs was verified as MeDEGs to pancreatic cancer using *R/Bioconductor packages edgeR* and *limma*. GO and KEGG pathway enrichment analysis were performed to analyze the enrichment of MeDEGs in the GO terms and pathways associated with pancreatic cancer. A univariate Cox model was performed to evaluate the correlation between 5 MeDEGs and the survival of patients with pancreatic cancer. Survival curves were constructed using the Kaplan–Meier method. GSEA and protein–protein interaction (PPI) were performed to elucidate the BP of prognosis-related MeDEGs in pancreatic cancer. To our knowledge, limited studies have been performed in regard to the cumulative analysis for MeDEGs of pancreatic cancer using array data from multiple platforms. Hence, the current study aimed to identify the potential MeDEGs associated with pancreatic cancer development and prognosis by bioinformatics analysis, in a bid to enhance the understanding of the molecular mechanisms that underpin the pathogenesis of pancreatic cancer.

## Materials and Methods

### Microarray-Based Gene Expression Analysis

Pancreatic cancer-associated RNA expression profiles and clinical data were initially retrieved from the TCGA database. Both RNA-Seq and clinical data were downloaded using a data transfer tool (provided by GDC Apps)^1^. The RNA-sequence count data between 178 pancreatic cancer samples and 4 normal samples, were merged into a matrix file using a Perl script^2^. The genome-build GRCh38.p13 version annotation file subsequently applied to transform the ensemble ID of the sample. The ensemble ID not included in the database was excluded, with the gene expression matrix subsequently obtained. The downloaded clinical data contained 185 cases. Illumina human methylation 450K BeadChip data were also downloaded from the TCGA database, including 185 pancreatic cancer samples and 10 normal samples. The original data pertaining to the methylation was composed of a β value (the ratio between the intensity of methylation probe and the intensity of total probe). The mean β value of all sites related to the gene was calculated and expressed as the average gene methylation value. The average methylation and expression data of one gene were then combined for Spearman’s rank correlation coefficient analysis. Due to the availability of the public data in the TCGA database, the study did not require ethical approval or informed consent.

### Identification of MeDEGs

Differentially expressed genes between 178 pancreatic cancer samples and 4 normal samples were identified using *R/Bioconductor package edgeR* with DMGs between 185 pancreatic cancer samples and 10 normal samples using *R/Bioconductor package limma* with | log2 fold change (FC)| > 0.5 and *p*-value < 0.05 as threshold. The correlation between gene expression and the average methylation value of the pancreatic cancer patients was analyzed using Spearman’s rank correlation coefficient analysis. Under the critical conditions of | log2 fold change (FC)| < −0.3 and *p*-value < 0.01, hypomethylated-high expression genes were obtained following the superimposition of upregulated and hypomethylated genes, with the hypermethylation-low expression genes obtained following the superimposition of both the downregulated and hypermethylated genes. The hypomethylation-high expression genes and hypermethylation-low expression genes were identified as MeDEGs. The MeDEGs were annotated via the CGC database^3^. Finally, a heatmap was plotted using *R/Bioconductor package pheatmap*.

### Gene Ontology and KEGG Pathway Enrichment Analysis of MeDEGs

In order elucidate the role of the identified MeDEGs in pancreatic cancer carcinogenesis and progression, the DAVID database^4^ was applied to perform a GO function enrichment analysis on MeDEGs. Default parameters were set for analysis. In the context of the entire human genome, functions enriched at *p* < 0.05 were considered to be important. In addition, the KEBAS database^5^ was adopted for KEGG pathway enrichment analysis on MeDEGs. The significance of the KEGG pathway was evaluated (*p* < 0.05).

### The Correlation Analysis Between MeDEGs and Prognosis of Pancreatic Cancer

According to the median value of MeDEG methylation, 185 patients with pancreatic cancer were divided into the hypermethylated and hypomethylated groups. The 185 patients were further divided into patients with hypermethylated and lowly expressed MeDEG (Hyper-LG) and patients with hypomethylated and highly expressed MeDEG (Hypo-HG) based on the median MeDEG methylation value and expression. The overall survival rate of two groups of patients was analyzed by univariate Cox model and survival curves were plotted by Kaplan–Meier method. A value of *p* < 0.05 was considered to be indicative of significant difference.

### Functional Prediction of Prognosis-Related MeDEGs

In order to deduce the potential biological function of the methylation status of prognosis-related MeDEGs, GSEA 3.0 software was applied to perform GSEA. The 185 pancreatic cancer samples from the TCGA database were divided into two groups (high vs. low) based on the median methylation level of prognosis-related MeDEGs. The annotated c2.cp.kegg.v6.2.symbols.gmt gene set in the Molecular Signature Database (MSigDB) was regarded as the reference gene set. The number and type of permutations were set to 1,000 and phenotype, respectively. NES > 1.4 and a value of *p* < 0.05 was considered to be reflective of significant difference.

### Construction of PPI Network of Prognosis-Related MeDEGs

In order to elucidate the molecular mechanism associated with the prognosis-related MeDEGs in pancreatic cancer, a PPI network was constructed using the STRING database 11.0^6^ with an interaction score >0.15 set as the threshold. The PPI network of the prognosis-related MeDEGs in pancreatic cancer was constructed in order to investigate the mechanism by which the prognosis-related MeDEGs influence the progress of pancreatic cancer through their interactions.

## Results

### Identification of MeDEGs in Pancreatic Cancer

**FIGURE 1:**
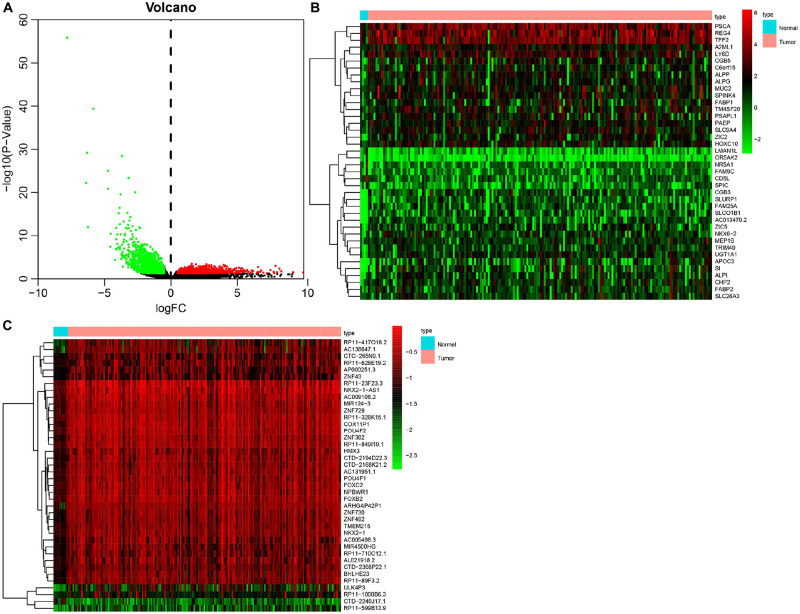
Identification of DEGs and DMGs in pancreatic cancer in TCGA database. **(A)** Volcano plot of DEGs between 178 pancreatic cancer samples and 4 normal samples. The red dots representing upregulated genes, green dots referring to downregulated genes, and black dots suggesting genes whose expression variation was less than 1. **(B)** Heatmap of top 40 DEGs between 178 pancreatic cancer samples and 4 normal samples. The ordinate on the left representing the cluster of DEGs, the ordinate on the right indicating the gene names, red suggesting the upregulated genes, and green referring to the downregulated genes. **(C)** Heatmap of DNA methylation signal of top 40 DMGs between 185 pancreatic cancer samples and 10 normal samples. The *x*-axis on the left referring to the cluster of DMG, the *y*-axis on the right representing the gene names, red suggesting the hypermethylated genes, and green indicating the hypomethylated genes.

**FIGURE 2:**
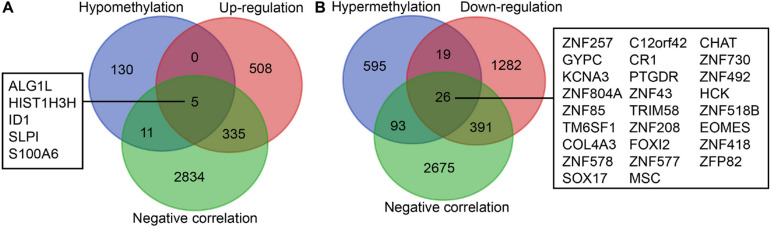
Identification of MeDEGs. **(A)** Identification of hypomethylated genes with upregulated expression. The three color ellipses in the figure represents the hypomethylated gene set, the gene set with upregulated expression and the gene set with significantly negative correlation between methylation and expression in pancreatic cancer samples from TCGA database, respectively. The middle part refers to the intersection of the three groups of data. **(B)** Identification of hypomethylated genes with downregulated expression. The three color ellipses in the figure indicates the hypermethylated gene set, the gene set with downregulated expression and the gene set with significantly negative correlation between methylation and expression in pancreatic cancer samples from TCGA database, respectively. The middle part suggests the intersection of the three groups of data.

**FIGURE 3:**
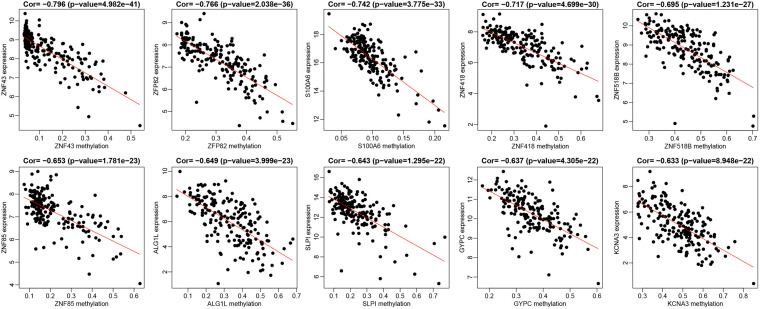
The top 10 MeDEGs with correlation coefficient. Spearman’s rank correlation coefficient analysis was performed between the methylation level (the *x*-axis) and the expression (the *y*-axis) of MeDEGs. The *x*-axis shows gene methylation in pancreatic cancer samples and the *y*-axis suggested gene expression in pancreatic cancer samples.

Initially, 2566 DEGs, including 848 upregulated genes and 1718 downregulated ones, between 178 pancreatic cancer samples and 4 normal samples were identified following our analysis of the RNA-seq data deposited in TCGA database. The volcano plot of the DEGs is depicted in Figure 1A, and the heatmap visualizing the ranking of the top 40 DEGs illustrated in Figure 1B. In addition to DEGs, 879 DMGs, including 733 hypermethylated genes and 146 hypomethylated ones, 185 pancreatic cancer samples and 10 normal samples were characterized. A heatmap visualizing the top 40 DMGs is illustrated in Figure 1C. In order to detect the genes driven by methylation, the correlation between gene expression and methylation data of pancreatic cancer patients was determined by Spearman’s rank correlation coefficient analysis. A significantly negative correlation was detected between the expressions of 3,185 genes and the average methylation levels. Finally, 31 MeDEGs were identified, including 5 hypomethylated/upregulated genes (ALG1L, HIST1H3H, ID1, SLPI, and S100A6) and 26 hypermethylated/downregulated genes (ZNF257, GYPC, KCNA3, ZNF804A, ZNF85, TM6SF1, COL4A3, ZNF578, SOX17, C12orf42, CR1, PTGDR, ZNF43, TRIM58, ZNF208, FOXI2, ZNF577, MSC, CHAT, ZNF730, ZNF492, HCK, ZNF518B, EOMES, ZNF418, and ZFP82). The Venn diagram and the list of MeDEGs are illustrated in Figure 2. The top 10 MeDEGs with the highest Spearman’s rank correlation coefficient are depicted in Figure 3. The 31 MeDEGs were annotated by the CGC database. Only the hypomethylated/upregulated gene ID1 and the hypermethylated/downregulated gene CR1 were both carcinogenic and tumor suppressor genes. The hypermethylated/downregulated gene HCK was just a tumor suppressor gene. The greater majority of the 31 MeDEGs had not been annotated in the CGC database.

### Gene Ontology Enrichment Analysis of MeDEGs

**FIGURE 4:**
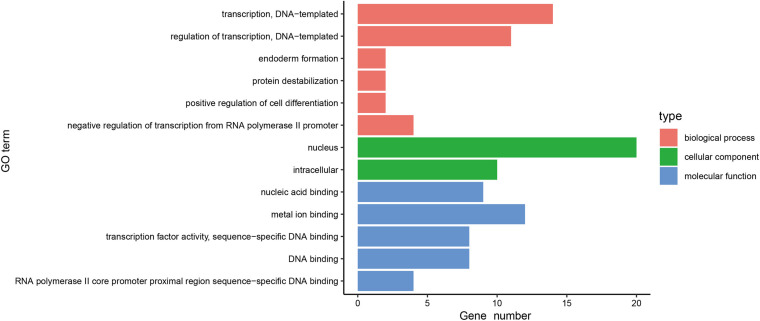
Gene ontology enrichment analysis of 31 MeDEGs in pancreatic cancer. GO enrichment analysis of 31 MeDEGs was performed using the DAVID database to perform on, and the significantly enriched GO terms were obtained (*p* < 0.05). The y-axis represents the GO terms on the significant enrichment, and the x-axis refers to the number of genes enriched on the GO terms.

In order to further investigate the function of the 31 MeDEGs in pancreatic cancer, the DAVID database was explored for GO enrichment analysis while the KOBAS database was used for the KEGG pathway enrichment analysis. GO enrichment analysis demonstrated that the MeDEGs related to BP were predominantly enriched in in GO terms including transcription, DNA-templated, regulation of transcription, DNA-templated, endoderm formation, protein destabilization, positive regulation of cell differentiation, and negative regulation of transcription from RNA polymerase II promoter, etc. (*p* < 0.05). MeDEGs associated with CC were most relevant to the nucleus (*p* < 0.05). In relation to MF, MeDEGs were primarily concentrated in nucleic acid binding, metal ion binding, transcription factor activity, sequence-specific DNA binding, DNA binding, RNA polymerase II core promoter proximal region sequence-specific DNA binding (*p* < 0.05) (Figure 4 and Table 1). KEGG pathway enrichment analysis displayed that MeDEGs were significantly enriched in certain pathways including that of malaria and legionnaires’ disease (*p* < 0.05) (Table 2). Taken together, the aforementioned results highlight the potentially crucial role played by the 31 screened MeDEGs in the development of pancreatic cancer.

**TABLE 1:** Gene ontology enrichment analysis of methylation-regulated differentially expressed genes associated with pancreatic cancer.

Categories	Term	Description	Genes name	*p*-value
BP	GO:0006351	Transcription, DNA-templated	ZNF43/ZNF208/ZNF85/ZFP82/FOXI2/ZNF518B/ID1/ZNF418/EOMES/ZNF578/ZNF492/ZNF730/ZNF257/ZNF577	1.66E-06
	GO:0006355	Regulation of transcription, DNA-templated	ZNF43/ZNF208/ZNF85/ZFP82/ZNF418/ZNF578/ZNF492/SOX17/ZNF730/ZNF257/ZNF577	4.48E-05
	GO:0001706	Endoderm formation	EOMES/SOX17	1.84E-02
	GO:0031648	Protein destabilization	ID1/SOX17	5.28E-02
	GO:0045597	Positive regulation of cell differentiation	EOMES/SOX17	5.58E-02
	GO:0000122	Negative regulation of transcription from RNA polymerase II promoter	ID1/EOMES/SOX17/MSC	9.84E-02
CC	GO:0005634	Nucleus	ZNF208/ZNF85/ZNF43/S100A6/ZNF518B/HCK/EOMES/MSC/ZFP82/FOXI2/ID1/ZNF418/ZNF578/ZNF492/SOX17/ZNF730/ZNF257/CHAT/HIST1H3H/ZNF577	7.08E-05
	GO:0005622	Intracellular	ZNF43/ZNF208/ZNF85/ZFP82/ZNF418/TRIM58/ZNF578/ZNF730/ZNF257/ZNF577	1.52E-04
MF	GO:0003676	Nucleic acid binding	ZNF43/ZNF208/ZNF85/ZFP82/ZNF418/ZNF578/ZNF730/ZNF257/ZNF577	1.42E-04
	GO:0046872	Metal ion binding	ZNF43/ZNF208/ZNF85/ZFP82/ZNF518B/ZNF418/ZNF578/ZNF492/ZNF730/ZNF257/ZNF577/ZNF804A	2.66E-04
	GO:0003700	Transcription factor activity, sequence-specific DNA binding	ZNF43/ZNF85/ZFP82/ID1/ZNF418/EOMES/MSC/ZNF577	7.81E-04
	GO:0003677	DNA binding	ZNF43/ZNF208/ZNF85/ZFP82/ZNF518B/EOMES/ZNF578/ZNF577	1.71E-02
	GO:0000978	RNA polymerase II core promoter proximal region sequence-specific DNA binding	ZNF85/ZNF492/ZNF730/ZNF257	2.05E-02

*BP, biological process; CC, cellular component; MF, molecular function.*

**TABLE 2:** The KEGG pathway terms enriched by methylation-regulated differentially expressed genes associated with pancreatic cancer.

Pathway ID	Description	Genes name	*p*-value
hsa05144	Malaria	CR1/GYPC	7.31E-04
hsa05134	Legionellosis	CR1	4.27E-02

### MeDEGs Are Associated With Prognosis in Patients With Pancreatic Cancer

**FIGURE 5:**
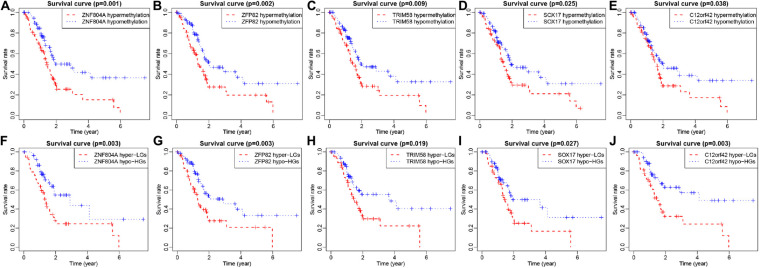
ZNF804A, ZFP82, TRIM58, SOX17, and C12orf42 were correlated with overall survival of patients with pancreatic cancer. **(A–E)** ZNF804A, ZFP82, TRIM58, SOX17, and C12orf42 ranked by median methylation level, and each patient with pancreatic cancer was scored on the basis of high or low methylation levels. **(F–J)** ZNF804A, ZFP82, TRIM58, SOX17, and C12orf42 ranked by the median of methylation level and their expressions, and each patient with pancreatic cancer was scored according to the high or low methylation levels as well as high or low expression. The *x*-axis represents the total survival time, and the *y*-axis suggests the survival function. Hyper-LG stands for hypermethylated and lowly expressed MeDEGs, while Hypo-HG refers to hypomethylated and highly expressed MeDEGs.

**FIGURE 6:**
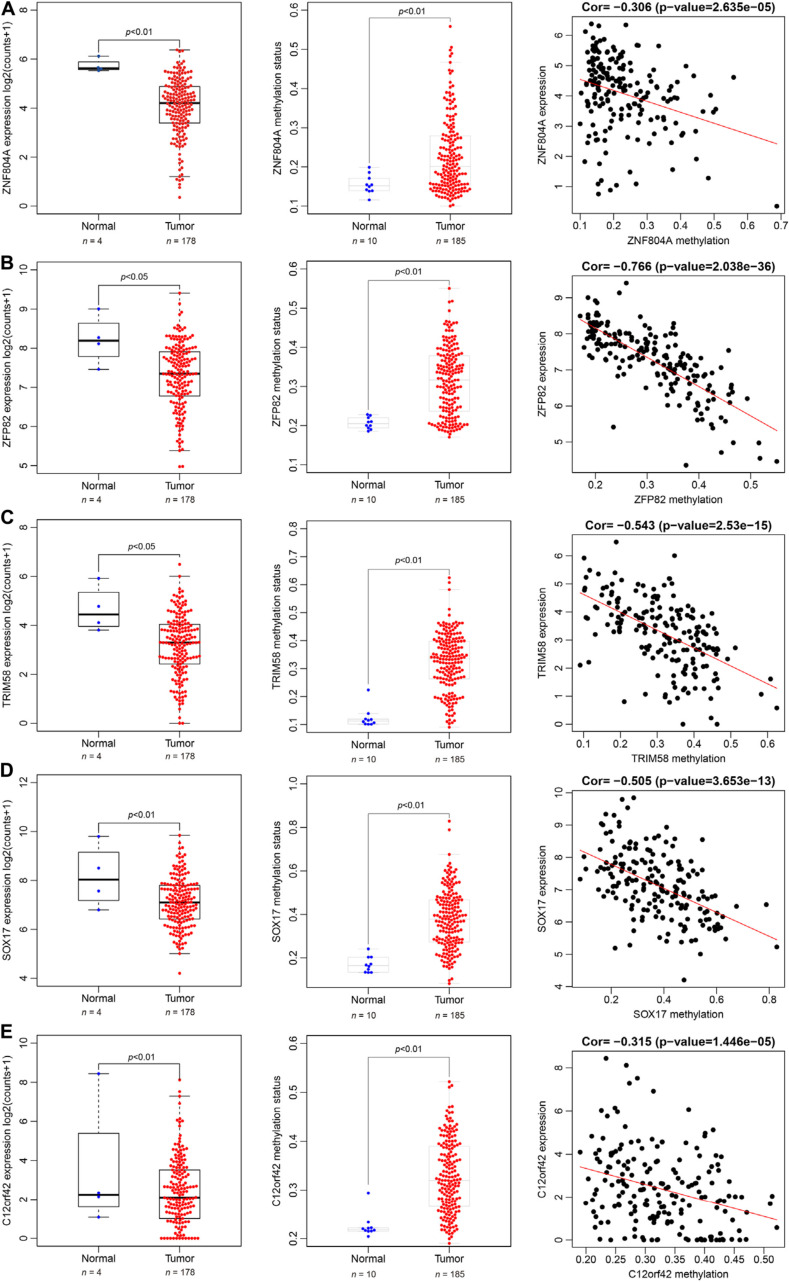
Spearman’s rank correlation coefficient analysis of expression and methylation level of ZNF804A, ZFP82, TRIM58, SOX17, and C12orf42. **(A–E)** The expression, methylation status and correlation of ZNF804A, ZFP82, TRIM58, SOX17, and C12orf42 analyzed, respectively using TCGA database.

The 185 patients with pancreatic cancer were divided into high and low methylation groups based the median MeDEGs methylation value. The patients were divided into hypermethylation low expression MeDEGs (Hyper-LG) group and hypomethylation high expression MeDEGs (Hypo-HG) groups based on the median methylation and expression of MeDEG value. Univariate Cox models were performed to evaluate the correlation between 31 MeDEGs and overall survival of the 185 patients. Kaplan–Meier curves illustrated the overall survival of patients with hypermethylation of Finger Protein 804A gene (ZNF804A), ZFP82 zinc finger protein (ZFP82), tripartite motif containing 58 (TRIM58), Sex-Determining Region Y-box 17 (SOX17) while the chromosome 12 open reading frame 42 (C12orf42) was lower than the patients with hypomethylation (Figures 5A–E). Next, comparison between the prognosis of patients with Hyper-LG and patients with Hypo-HG was performed. The results revealed that the overall survival of patients in the Hyper-LG group was notably lower than that of patients in the Hypo-HG group (Figures 5F–J). Expression, methylation level and Spearman’s rank correlation coefficient of ZNF804A, ZFP82, TRIM58, SOX17, and C12orf42 are all depicted in Figure 6.

### The Gene Enrichment Analysis of Prognosis-Related MeDEGs

**FIGURE 7:**
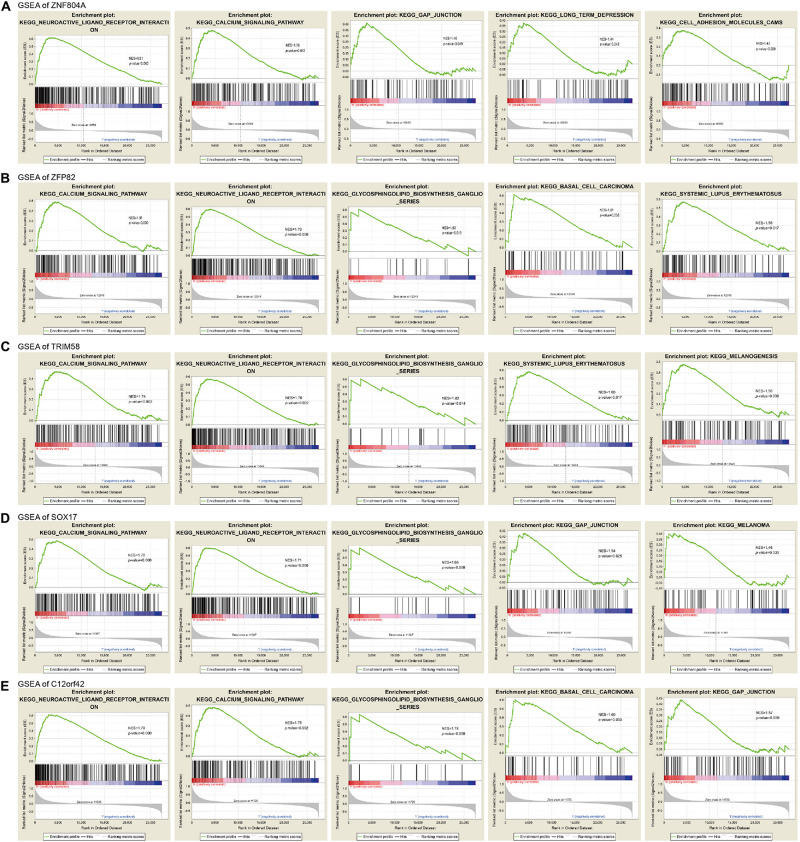
The gene enrichment analysis of ZNF804A, ZFP82, TRIM58, SOX17, and C12orf42. **(A–E)** The gene enrichment analysis of ZNF804A, ZFP82, TRIM58, SOX17, and C12orf42, respectively, using GSEA.

In order to clarify the biological function of the methylation status of MeDEGs associated with the prognosis of pancreatic cancer, the effect of MeDEG methylation status on the KEGG pathway was analyzed using GSEA 3.0 software. The top 5 GSEA results of MeDEGs are shown in Figure 7. Among which, the hypermethylation of ZNF804A, ZFP82, TRIM58, SOX17, and C12orf42 were all noted to be significantly associated with tumor-related pathways, including calcium signaling pathways, neuroactive ligand-receptor interaction, glycosynthetic ganglion series and intercellular junctions. Among which, the calcium signaling pathway and neuroactive ligand-receptor interaction was considered to be in the top 2 signaling pathways most significantly associated with ZNF804A, ZFP82, TRIM58, SOX17, and C12orf42 methylation level. These results demonstrated that certain genes including that of ZNF804A, ZFP82, TRIM58, SOX17, and C12orf42 may potentially be crucial regulatory factors in pancreatic cancer through pathways involving calcium signaling and neuroactive ligand-receptor interaction.

### Analysis of PPI Network of Prognosis-Related MeDEGs

**FIGURE 8:**
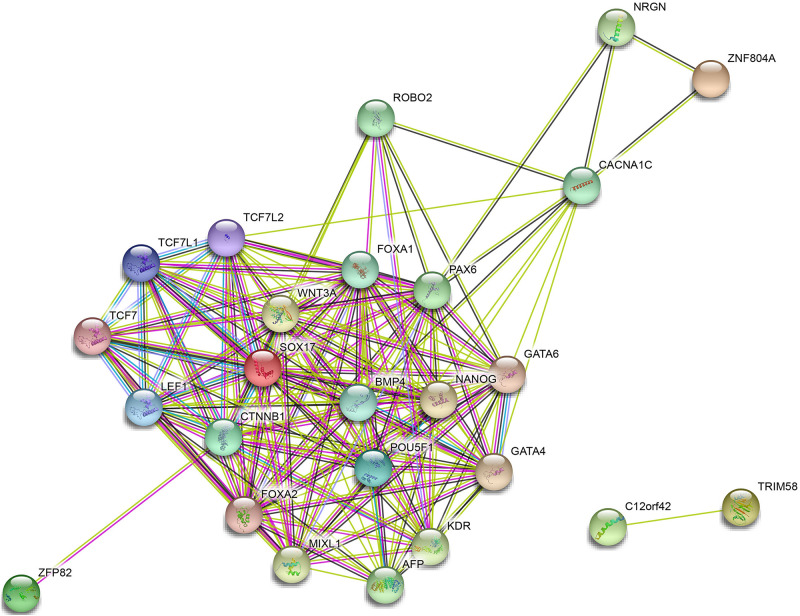
Protein–protein interaction network of ZNF804A, ZFP82, TRIM58, SOX17, and C12orf42. PPI of ZNF804A, ZFP82, TRIM58, SOX17, and C12orf42 analyzed by the STRING database.

A PPI network of ZNF804A, ZFP82, TRIM58, SOX17, and C12orf42 was constructed using the STRING database with PPI enrichment *p*-value: <1.0e-16 set as the threshold (Figure 8). The results illustrated that C12orf42 protein interacted with TRIM58 protein, SOX17 and ZFP82 protein simultaneously interacted with β-catenin 1 (CTNNB1). Furthermore, SOX17 was found to interact with a variety of proteins including kinase insert domain receptor (KDR), GATA binding protein-4 (GATA4), GATA binding protein-6 (GATA6), POU class 5 homeobox 1 (POU5F1). Moreover, ZNF804A was found to interact with neurogranin (NRGN), Calcium voltage-gated channel subunit α1C (CACNA1C) proteins. An indirect interaction was identified between SOX17 and the ZNF804A protein. Altogether, the results obtained provided evidence verifying demonstrating that these proteins could interact with each other to participate in the development of pancreatic cancer.

## Discussion

DNA methylation represents an epigenetic mechanism that regulates gene transcription, with various studies implicating aberrant DNA methylation in various cancers contributing to their progression (Mahmoud and Ali, 2019). Owing to the well documented fact that cancer progression requires many genetic alterations, aberrant DNA methylation play a key role in gene expression change associated with cancer progression and metastasis (Szyf, 2008). Recently, an increasing number of methylation-driven genes have been identified as promising candidate biomarkers in various cancers and related to cancer prognosis (Li et al., 2019; Liu et al., 2020). MeDEGs were screened by analyzing array data from the TCGA database, with the results suggesting their capacity to serve as prognostic markers regulated by promoter region methylation in cancer cell lines (Peng et al., 2020). Integrating the knowledge of epigenetics and gene expression contributes to the understanding of the mechanisms associated with cancer and may aid in providing a more accurate prognosis and verification of therapeutic targets (He et al., 2018). Bioinformatics analysis is a useful tool for the identification of MeDEGs in numerous cancers including hepatocellular carcinoma (Cai et al., 2019), cholangiocarcinoma (Mishra et al., 2020), and pancreatic cancer (Mishra and Guda, 2017; Liu et al., 2018; Cheng et al., 2019). Thus, attempts have been prudently made during the current study to identify MeDEGs in pancreatic cancer by means of bioinformatics analysis to investigate the potential related pathways associated with the progression of pancreatic cancer. In the current study, 5 hypermethylated/downregulated genes in pancreatic cancer were identified, including ZNF804A, ZFP82, TRIM58, SOX17, and C12orf42, all of which were found to be correlated with the poor survival of pancreatic cancer patients.

The Zinc finger protein family represent a series of small motifs that are abundantly expressed and play a crucial role in a variety of physiological and pathophysiological mechanisms. As a member of the zinc finger proteins family, ZNF804A has been linked to both schizophrenia and bipolar disorder (Squassina et al., 2019). Previous research has revealed that zinc finger proteins exert significant effects on cancer progression (Jen and Wang, 2016). The inhibition of PRDM14 has been shown to suppress tumor growth in pancreatic cancer (Taniguchi and Imai, 2018). ZFP82, also known as ZNF545, belongs to the Kruppel-related box zinc finger protein family, which is often down-regulated by promoter methylation serves as a tumor-suppressor gene in a variety of cancers (Cheng et al., 2012). The inhibition of ZFP82 though promoter methylation has been detected in colorectal cancer, which also possesses tumor-suppressing properties in the setting of colorectal cancer (Xiang et al., 2017). Previous literature has highlighted the downregulation of ZFP82 through promoter methylation in esophageal squamous cell carcinoma, which is associated with age, tumor stage as well as the prognosis of squamous cell carcinoma (Ye et al., 2019). Besides, tumor-specific methylation of ZFP82 has been implicated in multiple myeloma, with studies suggesting their potential as epigenetic biomarkers for the diagnosis of multiple myeloma as well as a promising target for specific treatment (Fan et al., 2016).

In addition, a previous investigation identified a link between low survival rate in gastric cancer patients and hypermethylated ZFP82. Thus, highlighting the potential of the methylated CpG site of the ZFP82 promoter as a clinical predictor of gastric cancer prognosis (Deng et al., 2015). SOX17 is a transcription factor that involved in regulating the formation and development of primitive endoderm and primitive germ cells (Tan et al., 2019). SOX17 promoter methylation has been suggested to offer crucial information for cancer prognosis. Furthermore, SOX17 has been reported to be highly methylated in non-small cell lung cancer which correlates with the survival time of patients with this disease (Balgkouranidou et al., 2016). The methylation of SOX17 has also been linked with poor survival of breast cancer (Fu et al., 2015).

As a member of the tripartite motif protein (TRIM) family, TRIM58 has been reported to function as a tumor-suppressor gene in many cancers including colorectal cancer (Liu et al., 2018) and gastric cancer (Fu et al., 2015). Besides, a decrease in the expression of TRIM58 via hypermethylation has been strongly associated with cancer progression in patients with early stage lung adenocarcinoma (Kajiura et al., 2017). Xu et al. (2019) demonstrated that MeDEGs such as TRIM58 exerts great distinct influence on the development of pancreatic cancer via bioinformatics analysis.

Furthermore, our results displayed that these five genes may play crucial regulatory roles in pancreatic cancer through pathways such as calcium signaling and neuroactive ligand-receptor interaction. Calcium signaling (Sterea and El Hiani, 2020) and neuroactive ligand-receptor interaction (Wei et al., 2012) have been linked with cancer-related pathways. Interestingly, hypermethylation in calcium-signaling pathway has been identified in various cancers while MeDEGs involved in the calcium signaling pathway may be an effective strategy for cancer treatment (Wang et al., 2017).

## Conclusion

The key findings of the current study highlights ZNF804A, ZFP82, TRIM58, SOX17, and C12orf42 as hypermethylated/downregulated genes in pancreatic cancer associated with the development and progression of pancreatic cancer through their modulation of specific pathways. However, the role of C12orf42 in pancreatic cancer requires further experimental validation. Greater detail is required in order to further identify the fundamental molecular mechanisms to confirm the results of the candidate genes and pathways identified. Nevertheless, these results will help us to identify significant therapeutic targets and biomarkers for pancreatic cancer and enhance the understanding of the cumulative role of epigenetic mechanisms in the pathogenesis of pancreatic cancer.

## Data Availability Statement

The original contributions presented in the study are included in the article/supplementary material, further inquiries can be directed to the corresponding author.

## Ethics Statement

Due to the availability of public data in the TCGA database, this study did not require ethical approval or informed consent.

## Author Contributions

HS: conceptualization. GH: investigation and validation. RX: data collection and visualization. CZ: writing – original draft. HS and CZ: writing – review and editing. All authors read and approved the final manuscript.

## Conflict of Interest

The authors declare that the research was conducted in the absence of any commercial or financial relationships that could be construed as a potential conflict of interest.

## Footnotes

1 https://tcga-data.nci.nih.gov/

2 http://www.perl.org/

3 https://cancer.sanger.ac.uk/census

4 https://david.ncifcrf.gov/

5 https://www.cbi.pku.edu.cn/resource/index.htm

6 http://STRING-db.org/

## Abbreviations

MeDEGs: methylated-differentially expressed genes
DEGs: differentially expressed genes
DMGs: differentially methylated genes
PPI: protein–protein interaction
SOX17: Sex-Determining Region Y-box 17
C12orf42: chromosome 12 open reading frame 42
ZFP82: ZFP82 zinc finger protein
TRIM58: tripartite motif containing 58
TCGA: The Cancer Genome Atlas
KEGG: The Kyoto Encyclopedia of Genes and Genomes
GO: gene ontology
CC: cellular component
MF: molecular function
BP: biological process
GSEA: gene set enrichment analysis
Hyper-LG: hypermethylated and lowly expressed MeDEG
MSigDB: Molecular Signature Database
CTNNB1: simultaneously interacted with β -catenin 1
KDR: kinase insert domain receptor
GATA4: GATA binding protein-4
GATA6: GATA binding protein-6
POU5F1: POU class 5 homeobox 1
NRGN: neurogranin
CACNA1C: calcium voltage-gated channel subunit α 1C
TRIM: tripartite motif protein.
